# Promoting psychology to students: embracing the multiplicity of research foci and method

**DOI:** 10.3389/fpsyg.2013.00774

**Published:** 2013-10-21

**Authors:** Clare S. Rees

**Affiliations:** School of Psychology and Speech Pathology, Curtin UniversityPerth, WA, Australia

**Keywords:** psychology, education, research methods

## Abstract

In order for the discipline of psychology to continue to thrive it is imperative that future students are effectively recruited into the field. Research is an important part of the discipline and it is argued that the nature of psychological research is naturally one of multiplicity in topic and methodology and that promoting and highlighting this should be considered as a potentially effective recruitment strategy. In this study, a snap-shot of current research topics and methodologies was collected based on published papers from one typical academic psychology department in Australia. Fifty articles published in the period 2010–2013 were randomly selected and then grouped using content analysis to form topic clusters. Five main clusters were identified and included: Grief and Loss; Psychopathology; Sociocultural Studies; Attachment and Parenting; and Developmental Disorders. The studies spanned the full spectrum of research methodologies from quantitative to qualitative and had implications for assessment practices, diagnosis, prevention and treatment, education, and policy. The findings are discussed in terms of the unique characteristics of psychology as a discipline and how this diversity ought to be utilized as the main selling point of the discipline to future students.

## Introduction

The discipline of Psychology has been described as a “human science” because it intercepts both the field of humanities as well as the traditional sciences. However, despite this overlap, Psychology is a unique and separate discipline. It seeks to address questions about human nature by utilizing many of the empirical methods of the physical sciences yet at the same time the complexity of studying human nature makes psychological research inherently subjective and selective. Research methods within psychology cover a broad spectrum such as basic laboratory-based research, case studies, correlational studies and mixed methodologies. In the late 1970s a philosopher of science, Wilhelm Dilthey referred to the multiplicity of research methods employed in psychology as a necessary aspect of the discipline:

Psychology depends on different approaches compensating for each other's defects …. It seeks entry into mental life through many gates (Dilthey, 1976, p. 93).

In many ways the research that underpins the study of psychology is both linear and circular and the tension that exists between acknowledging the importance of the scientific method but at the same time acknowledging the notion that much of the field is concerned with phenomena that is largely outside of the realm of measurement creates a dialectic. This can make the study of psychology both unsettling/off-putting and attractive at the same time. In terms of attracting potential students to the discipline it may be important to use this dialectic as a selling point as opposed to attempting to falsely and artificially force psychology into one category or another, e.g., science or humanities.

Students new to the field of psychology tend to have false ideas as to what the general subject matter involves. For example, Rowley et al. (2008) surveyed 169 honors students from a research active university in the United Kingdom. They found that in their first year of studying psychology, students believed that the subject was primarily concerned with uncovering underlying truths about human nature. It wasn't until the students reached second or third year that they were more likely to understand and appreciate the diversity of focus and perspectives in the discipline.

Interestingly, most of the published literature relating to research in psychology is concerned primarily with debating approaches to research methodology. For example, Breen and Darlaston-Jones (2010) argued that psychological research is dominated by positivism and a narrow preference for quantitative research at the expense of other equally rigorous forms of enquiry. They go on to propose that increasing the diversity within psychology “would enhance the applicability and relevance of the profession to addressing social issues that would lead to meaningful social change, which would certainly increase the ‘real-world' impact of the research” (p.71). Some authors have argued that the focus on research methodologies in psychology has been an attempt to unify an otherwise fragmented discipline by searching for a unified research method. Henriques (2013) has suggested that this is a flawed endeavor and has instead proposed a “Unified Theory” which focuses on providing a conceptual map of the full breadth of psychological enquiry.

However, in order to accurately construct such a “map” it is first necessary to obtain a clear picture of the type of research currently being conducted within the discipline. A recent exploration of published studies from 2007–2013 using one of the leading psychology search engines, *Psych Info*, did not uncover one article providing an overview of representative research topics in psychology. For academic departments to remain financially viable it is vital that students are effectively recruited. For psychology this involves the use of effective marketing strategies that will generate interest in the topic but at the same time provide accurate information as to the nature of the course to be studied. With this in mind the purpose of the current paper was to investigate the current status of research in psychology in a typical academic psychology department. Focusing on one department was seen as a starting point for a subsequent larger study encompassing several Australian universities. However, as an initial step in the research process and because no other comparable studies could be identified, a purposive sample was selected. Information as to the topics and research methodologies currently being undertaken will help provide an accurate profile of the scope of psychology needed to assist in the effective recruitment of future students to the field.

## Materials and methods

As the purpose of the research was to create a representative snap-shot of published research in the field, inductive content analysis was the method employed. All published, peer-reviewed articles produced from the School of Psychology, Curtin University, Australia in a three year period (2010–2013) were collected. Next, 50 articles were randomly selected from the total list of published articles. These articles were then examined and grouped into clusters based on the topic of the research. Articles that did not fall into a major cluster were assigned minor clusters. Each article was also labeed as either quantitative, qualitative, mixed-method or review. Clusters groups were determined by the author who is an experienced research active academic. In order to ensure the trustworthiness of the data, member checks of the resultant cluster groups were conducted with the authors of the papers.

## Results

Five dominant clusters emerged. Each is described in detail below. Table 1 outlines the articles and indicates the associated research methodology used.

**Table 1:** **Research Methodologies and Samples of the included studies by theme**.

**Research theme**	**Research method**
Grief and loss	Aoun et al. (2012): Review
	Aoun et al. (2013): Quantitative
	Breen and O'Connor (2010): Qualitative
	Bentley et al. (2012): Quantitative
	Breen (2012): Review
Psychopathology	Egan et al. (2011): Quantitative
	Egan et al. (2012b): Review
	Egan et al. (2012a): Quantitative
	Fitt and Rees (2012): Quantitative
	Kingdon et al. (2012): Quantitative
	Lee and Rees (2011): Qualitative
	Mazzucchelli (2010): Review
	Priddis and Wallace (2011): Qualitative
	Rees and Anderson (2013): Review
	Steele et al. (2013): Quantitative
Social cultural	Bennett et al. (2012): Qualitative
	Di Ciano et al. (2010): Qualitative
	Dzidic and Green (2012): Mixed Method
	Garvey (2011): Review
	Hofmeester et al. (2012): Qualitative
	Prandl et al. (2012): Review
	Rooney et al. (2012): Quantitative
	Syme et al. (2012): Mixed Method
Parenting and attachment	Howieson and Priddis (2011): Qualitative
	Priddis and Wells (2010): Qualitative
	Priddis and Wallace (2011): Qualitative
	Priddis and Howieson (2012): Quantitative
	Sanders and Mazzucchelli (2013): Review
Developmental disorders	Haynes et al. (2013): Quantitative
	Kidd and Kaczmarek (2010): Qualitative
	Pannekoek et al. (2012): Quantitative
	Pearsall-Jones et al. (2010): Review
	Pearsall-Jones et al. (2011): Mixed Method
	Povee et al. (2012): Mixed Method
	Sheikhi et al. (2013): Quantitative
	Wilson et al. (2013): Quantitative

### Grief and loss

The studies in this cluster covered the full breadth of research from preliminary qualitative studies (Breen and O'Connor, 2010), intervention studies (Bentley et al., 2012) through to those concerned with policy and practice (Aoun et al., 2012, 2013; Breen, 2012). For example, the study by Breen and O'Connor (2010) explored the experience of grief amongst 21 adults whose family member had been killed in a traffic crash. Participants were drawn from 16 families and were aged between 24 and 71 years. Semi-structured interviews were conducted and analyzed from a grounded theory perspective. Participants described their grief as being “silenced” on a number of levels. Overall, the authors concluded that participants were communicating a “resistance to the dominant grief discourse” as their experiences did not align with the predominant view of grief as being a linear, staged-based process. Participants indicated a preference for a more holistic rather than symptom-focused model of care.

Aoun et al. (2012) conducted a review of bereavement services in palliative care and argued for a public health approach to the provision of such services. A public health approach would tailor provision of services according to a three-tiered model. In this model, interventions are either universal, selective or indicated and thus differ in intensity. At the universal level all bereaved are provided with information about bereavement, at the selective level those assessed as at-risk of developing complex needs are provided with non-professional support such as community support groups and at the indicated level those with complex needs are provided with specialist bereavement supports. The review strongly links recommendations to empirical studies showing that many people manage their grief effectively without the need for invasive interventions. As these studies in grief and loss reveal, a number of different research methodologies are employed form basic research through to translational research.

### Psychopathology

The studies included in this cluster ranged from those looking at particular psychological disorders such as depression (Mazzucchelli, 2010), eating disorders (Joyce et al., 2012; Egan et al., 2013), obsessive-compulsive disorder (OCD) (Lee and Rees, 2011; Fitt and Rees, 2012; Kingdon et al., 2012; Rees and Anderson, 2013), and substance abuse (Priddis and Wallace, 2011; Bright et al., 2013) through to those studying transdiagnostic factors considered to be pivotal in the development and maintenance of psychological disorders, such as perfectionism (Egan et al., 2011, 2012a, b; Steele et al., 2013). The studies included in this group had a diverse range of methodologies. The study by Joyce et al. (2012) studied the relationship between perfectionism and eating disorder pathology using structural equation modeling. A community sample of 202 women completed a number of questionnaires assessing conditional goal setting, self-oriented perfectionism and eating pathology. The resulting model indicated that shape and weight overvaluation and conditional goal-setting mediated the relationship between self-oriented perfectionism and eating pathology.

The study by Lee and Rees (2011) was a qualitative investigation of the treatment experiences and preferences of patients diagnosed with OCD. In particular, this study sought to determine if the experience of exposure therapy (the gold-standard psychological treatment for OCD) was as aversive as had been reported anecdotally. Thematic analysis of individual responses to semi-structured interviews indicated that participants found the treatment to be “challenging and stressful” but that the level of distress was moderated by the level of therapist support and explanation provided. Overwhelmingly, the participants were in support of the therapy acknowledging that it had been the necessary ingredient to their symptom improvement.

### Social cultural studies

This research cluster included studies of indigenous Australians and mental health (Garvey, 2011; Bennett et al., 2012; Prandl et al., 2012), the mental health needs of immigrants (Di Ciano et al., 2010; Rooney et al., 2012) and social cultural influences on the environment (Dzidic and Green, 2012; Hofmeester et al., 2012; Syme et al., 2012). Rooney et al. (2012) constructed a measure to assess the level of ethnic identification of adults from diverse ethnic backgrounds. A second aim of developing the measure was to assess the level of ethnic identification with the host culture. They included 275 adults from ethnically diverse backgrounds who responded to 50 initial statements. Factor analysis of responses produced three factors: (1) Pride in ethnic background and language, (2) Liking for traditional and social activities of my ethnic group, and (3) Sense of belonging to this (host) country. The authors then tested the model on a further sample of 1007 adults. A final two-factor model was derived; (1) Ethnic Identity and (2) Sense of Belonging to host country.

Dzidic and Green (2012) utilized a mixed-method approach to studying community acceptance of non-potable groundwater use and sustainable urban design in Perth, Australia. The authors utilized a computer-based photo-elicitation survey to ascertain respondents acceptance of various degrees of aesthetic degradation, for example, discoloration of toilets due to use of groundwater. Eleven in-depth interviews were also conducted and the overall finding was that participants were accepting of some level of aesthetic degradation however social norms and pressures to “keep up appearances” is an obstacle to uptake of sustainable urban design.

### Parenting and attachment

This cluster included intervention studies for improving parent-infant relationships and general parenting skills (Priddis and Wells, 2010; Sanders and Mazzucchelli, 2013), studies specifically investigating affect regulation amongst parents (Howieson and Priddis, 2011; Priddis and Wallace, 2011) and studies of attachment patterns (Priddis and Howieson, 2012). The study by Priddis and Howieson (2012) compared attachment patterns with concurrent behavioral ratings in a sample of preschool children. The sample included 136 mother-infant dyads from diverse socio-economic backgrounds. No association between attachment patterns and behavioral ratings was found at preschool age, however follow-up of the same sample 7 years later revealed an association between compulsively insecure attachment patterns and levels of depression.

Sanders and Mazzucchelli (2013) published a review article that focused on the importance of integrating a self-regulation component to parent training programs as a way of improving parenting practices. They argued that parents with a strong self-regulatory capacity would be in a better position to promote self-regulation skills in their children.

### Developmental disorders

Studies in this category covered developmental coordination disorder (DCD) (Pearsall-Jones et al., 2010, 2011; Pannekoek et al., 2012; Wilson et al., 2013), autism (Kidd and Kaczmarek, 2010), intellectual disability (Povee et al., 2012; Haynes et al., 2013), and attention deficit hyperactivity disorder (ADHD) (Sheikhi et al., 2013). Pearsall-Jones et al. (2010) reviewed the etiological pathways of DCD and ADHD and argued that although these developmental disorders tend to overlap and have some shared features such as prevalence and ratio of males to females with the disorders, they have different developmental pathways. They suggest that the causal pathways of developmental disorders are best considered a combination of genetic, epigenetic and environmental factors and that these factors evolve over time.

The study by Povee et al. (2012) utilized a mixed-method approach to study the impact on families of raising a child with Down's Syndrome. The study included 224 caregivers of children aged 4–25 years. The quantitative aspect of the study investigated the relationship between amount of maladaptive and autism-spectrum behaviors on family functioning. Although greater levels of these behaviors was associated with poorer family functioning, on the whole family functioning and marital adjustment did not differ from levels found in families with typically developing children.

### Other minor themes

Several other minor research themes were observed and further illustrate the diversity in topic range within the discipline. These themes include; Computers and Online Environments (Burns and Roberts, 2013; Jerejian et al., 2013); music preferences (Yeoh and North, 2012); teaching and supervision (Grant et al., 2012; Schneider and Rees, 2012; Schofield and Grant, 2013) maternal stress and pregnancy issues (Doyle et al., 2013; Li et al., 2013) and forensic psychology (Halse et al., 2012; Mackenzie et al., 2012; Spiranovic et al., 2012).

## Discussion

The analysis of collected published articles for the period 2010–2013 revealed a wide range of topic and an even spread of research methodologies. The collection of studies illustrates the diversity of research foci in the discipline of psychology. The widely held notion that psychological research is restricted to studies of psychological disorder at the individual level is directly refuted by the results of this review. A further important observation is that research methodologies were very evenly spread with approximately half of the studies being quantitative and half qualitative or mixed methodology. Qualitative methodologies have gained increasing favor and the results of this study support the popularity of this approach. The results of this study suggest that the discipline is beginning to utilize a more even blend of research methodologies which is in line with the suggestions made by Breen and Darlaston-Jones (2010).

It should be acknowledged that the present investigation is limited to the study of one psychology department in Australia. In order to capture a more comprehensive picture of the state of research in psychology this study requires replication and extension with a broader representation of the field. However, it offers an important first step in assessing the state of play in the discipline.

Psychology as a research field is thriving internationally but in order for it to continue to grow it is imperative to attract enthusiastic and capable researchers to the field. The current findings indicate that marketing initiatives in universities should consider showcasing examples of published research in order to clearly communicate the diversity that is possible when electing to study this field. As highlighted in this review, research in psychology crosses the full spectrum of human functioning and behavior. The impacts of psychological research can be seen in policy and practice developments, in community education, and in prevention and treatment. Rather than struggle to fit a round peg in a square hole, it may be more fruitful for efforts to focus on describing the discipline in terms of its rich topic diversity and methodological range.

Over the years psychology could be described as having an “identity crisis”; not fitting comfortably into either the pure sciences or the humanities but falling somewhere in-between. Interestingly, pioneers in the field of psychology such as Windt, James, Binet, and Freud, all used multiple methods, both quantitative and qualitative. It seems that contemporary psychology continues in this tradition.

### Conflict of interest statement

The author declares that the research was conducted in the absence of any commercial or financial relationships that could be construed as a potential conflict of interest.
